# Extending the global landscape of Bruck syndrome: Case series of Indonesian and Ukrainian patients with PLOD2 pathogenic variants and literature review

**DOI:** 10.1016/j.bonr.2026.101938

**Published:** 2026-07-07

**Authors:** Devina Afraditya Paveta, Agustini Utari, Ferdy Kurniawan Cayami, Alessandra Maugeri, Aare Märtson, Sulev Kõks, Andrii Pashenko, Sergii Khmyzov, Elisabeth Marelise W. Eekhoff, Katre Maasalu, Dimitra Micha, Lidiia Zhytnik

**Affiliations:** a Master of Biomedical Science, Faculty of Medicine, Universitas Diponegoro, Semarang, Indonesia; b Center of Biomedical Research (CEBIOR), Faculty of Medicine, Universitas Diponegoro, Semarang, Indonesia; c Department of Pediatrics, Kariadi Hospital/Faculty of Medicine, Universitas Diponegoro, Semarang, Indonesia; d Department of Human Genetics, Amsterdam Reproduction and Development, Amsterdam Movement Sciences, Amsterdam UMC Location Vrije Universiteit, Amsterdam, the Netherlands; e University of Tartu, Department of Orthopaedics, Tartu, Estonia; f Tartu University Hospital, Clinic of Orthopaedics, Tartu, Estonia; g Personalised Medicine Centre, Murdoch University, Murdoch, WA, Australia; h Perron Institute for Neurological and Translational Science, Nedlands, WA, Australia; i Sytenko Institute of Spine and Joint Pathology, National Academy of Medical Science of Ukraine, Kharkiv, Ukraine; j Department of Endocrinology and Metabolism, Amsterdam Reproduction and Development, Amsterdam Movement Sciences, Amsterdam UMC location Vrije Universiteit, Amsterdam, the Netherlands

**Keywords:** Congenital bone fragility, Bruck syndrome, Lysyl hydroxylase 2, PLOD2 gene, Bone fracture

## Abstract

Bruck syndrome type 2 (BRKS2) is a rare disorder marked by congenital joint contractures and bone fragility, caused by variants in PLOD2, which encodes lysyl hydroxylase 2 essential for collagen stability. We report the first genetically confirmed BRKS2 cases from Indonesia and Ukraine, both showing fractures, skeletal deformities, and contractures. The Indonesian patient had compound heterozygous variants, while the Ukrainian patient had a homozygous missense variant, expanding the phenotypic and geographic spectrum of the disorder.

## 1 Introduction

Bruck Syndrome (BRKS) is an ultra-rare autosomal recessive skeletal disorder characterized by the combination of congenital bone fragility and joint contractures (arthrogryposis) (Kaneto et al., 2016; Gistelinck et al., 2021). The estimated prevalence of BRKS is less than 1 in 1,000,000, with just over 60 cases reported in the literature to date. While BRKS shares several clinical features with osteogenesis imperfecta (OI), including low bone mass, perinatal fractures, skeletal deformities, and short stature, it can be distinguished by the presence of progressive joint contractures, elbow pterygia, and clubfeet (Leal et al., 2018; Puig-Hervas et al., 2012). Unlike classical OI, it was previously assumed that individuals with BRKS typically do not exhibit blue sclerae, dentinogenesis imperfecta (DI), or hearing loss. However, recent studies have expanded the phenotypic spectrum of BRKS to include camptodactyly, orodental anomalies, facial dysmorphism, congenital heart defects, and varying degrees of intellectual development, highlighting the phenotypic heterogeneity of the condition (Table 1) (Caparros-Martin et al., 2017; Lv et al., 2018; Mumm et al., 2020; Otaify et al., 2016; Terajima et al., 2022).

**Table 1:** Geographic, genetic and clinical landscape of patients with Bruck Syndrome type 2 (BRKS2) and (likely) pathogenic variants in the *PLOD2* gene (NM_182943.3) reported in the literature.

Country of ethnic origin	Number of patients/families	Zygosity	Pathogenic variants	Variation ID (ClinVar)	Exon	Pathogenic variant type	Clinical Features	Reference
Indonesia (Case 1)	1/1	Compound heterozygous	c.880-1G > C, p.?	NA	8i	Splice site, Frameshift	Blue sclerae, Osteopenia, joint contractures, joint hypermobility, recurrent fractures, skeletal deformities, Wormian bones, kyphosis, gross motor developmental delay, microcephaly, severe underweight, short stature.	Present article
			c.1559dupC, p.(Val523Cysfs*7)	41,424	14			
Ukraine (Case 2)	1/1	Homozygous	c.1958C > G, p.(Pro653Arg)	1,705,607	18	Missense	Grey sclera, joint contractures (arthrogryposis), recurrent fractures, skeletal deformities, pectus excavatum, kyphoscoliosis, cleft palate, hypotrophy	Present article
Turkey	1/1	Homozygous	c.1856G > A, p.(Arg619His)	7643	18	Missense	Blue-greyish sclerae. Contractures were present at the wrists, and there were bilateral clubfeet. Bilateral inguinal hernias were also diagnosed. Fractures and deformities	23
Lebanon	1/1	Homozygous	chromosome 3q22.1q24 0.07 Mb deletion (partial deletion of the *PLOD2* gene)	NA	NA	Multiexon deletion	Prenatal fractures, severe bone deformities, contractures, skin tethering, facial dysmorphism, congenital cardiac disease and pulmonary hemorrhage. Died at the age of 6 weeks.	25
Afghanistan	2/1	Homozygous	c.2060 A > G, p.(His687Arg)	988,314	19	Missense	Older sister: multiple contractures in her elbows, hips, knee and ankle joints, severe kyphoscoliosis, and a non-developed left patella, fractures, deformities, Younger sister: Grey-white sclerae, brachial plexus injury, torticollis, and stiff hips, fractures, Wormian bones, no contractures, skeletal deformities.	3
Kurdish	2/1	Homozygous	c.1865G > T, p.(Gly622Val)	7642	18	Missense	NA	27
Palestine	1/1	Homozygous	c.1764G > T, p.(Trp588Cys)	988,376	17	Missense	White sclera, no joint contractures, fractures.	3
India	1/1	Homozygous	c.797G > T, p.(Gly266Val)	2,076,023	8	Missense	White sclera, Limbs, bilateral knee joints, elbow joints, wrist joints and fingers contractures, elbows pterygia, deformities, overcrowded malformed teeth, normal hearing, pectus carinatum, severe kyphoscoliosis, mild bilateral clubfoot, and overriding of second and third digits of the right foot	20
India	1/1	Homozygous	c.648C > A, p.(Cys216*)	1,696,826	6	Nonsense	Prenatal lethality	24
China	1/1	Compound heterozygous	c.2038C > T, p.(Arg680^⁎^)	374,012	19	Nonsense	Prenatal lethality, miscarriages	12
			c.191_201 + 3delATACTGTGAAGGTA, p.(Ter64Cysfs^⁎^12)	NA	2	Frameshift		
China	1/1	Compound heterozygous	c.202G > T, p.(Val68Phe)	NA	3	Missense, Nonsense	Bruck syndrome,NA	28
			c.1042dupT, p.(Asp348*)	NA	10			
China	1/1	Compound heterozygous	c.1624delT, p.(Tyr542Thrfs*18)	NA	15	Frameshift Missense	White sclera, Wormian bones, left pes equinovarus and mild limitation of joint movement of knees and right elbow, recurrent fractures, Camptodactyly, kyphoscoliosis, deformities	19
			c.1880 T > C, p.(Val627Ala)	NA	18			
China	1/1	Compound heterozygous	c.503-2 A > G, p.?	NA	4i	Splice site, Missense	White sclera, congenital contracture of left elbow, fractures, kyphoscoliosis, Wormian bones, generalized osteoporosis, thoracic cage collapse, deformity of pelvis,.	6
			c.1138C > T, p.(Arg380Cys).	1,329,068	11			
China	1/1	Compound heterozygous	c.1153 T > C, p.(Cys385Arg)	NA	11	Missense	Blue-grey sclera, congenital contracture of knees, ankles, and elbows, recurrent fractures, Camptodactyly, Wormian bones in cranium, metaphyseal enlargement of distal femur	6
			c.1982G > A, p.(Gly661Asp)	1,366,020	18	Missense		
China	1/1	Compound heterozygous	c.2038C > T, p.(Arg680*)	374,012	19	Nonsense,	White sclera, congenital contracture of elbows and left ankle, fractures, Camptodactyly, Wormian bones,	6
			c.1138C > T, p.(Arg380Cys)	1,329,068	11	Missense		
Australia	3/1	Homozygous	c.1886C > T, p.(Thr629Ile)	7641	18	Missense	2 deceased prenatally, 1 deceased at the age of 2	27
Egypt	1/1	Homozygous	c.1856G > A, p.(Arg619His)	7643	18	Missense	Bulging chest (arrow), anterior bowing of legs (arrowhead), flexion contractures of both elbows, wrists, and knees	4
Egypt	2/1	Homozygous	c.1856G > A, p.(Arg619His)	7643	18	Missense	Scoliosis at dorsal region (arrow). Note also old healed rib fracture with callus formation (arrowhead)	4
Egypt	1/1	Homozygous	c.1559dupC, p.(Val523Cysfs^⁎^7)	41,424	14	Frameshift	Camptodactyly, contractures at hands, wrists, elbows, knees, and bilateral talipes. Arrow designates pterygium at axilla.	4
Egypt	1/1	Homozygous	c.1559dupC, p.(Val523Cysfs^⁎^7)	41,424	14	Frameshift	Loss of bone modeling with fractures and bowing of femora and tibia with malunited fracture of left femur	4
Egypt	1/1	Homozygous	c.1358 + 5G > A, p.?	NA	12i	Splice site	Recurrent fractures, bone deformities, no contractures, Partial ptosis of eyelids, white sclera, short philtrum, macrostomia, thick lips, thick upper ear helices, and short neck were noted, dislocation of left shoulder joint, prominent clavicles, arachnodactyly of fingers, prominent knee joints, anterior bowing of right leg, bilateral clinodactyly of 5th toes, deformed chest with flaring of ribs, winging of scapulae, and kyphoscoliosis	4
Egypt	1/1	Homozygous	c.1358 + 5G > A, p.?	NA	12i	Splice site	No congenital contractures, fractures, blue sclera, very mild hypotonia, wormian bones	5
Egypt	2/1	Homozygous	c.1828 T > C, p.(Trp610Arg)	NA	17	Missense	Greyish blue sclera, congenital joint contractures, bone deformities, kyphoscoliosis, and muscle wasting of lower limbs, recurrent fractures.	5
							Facial palsy, developmental delay, dilatation of ventricular system (one of probands)	
Egypt	1/1	Homozygous	c.1856G > A, p.(Arg619His)	7643	18	Missense	White sclera, multiple fractures, Bilateral hip, knee and ankle contractures, mild kyphosis, Arachnodactyly, hemangioma over left eye, café au lait patch over right knee	19
Egypt	1/1	Compound heterozygous	c.1280 A > G, p.(Asn427Ser)	807,467	12	Missense, Frameshift	White sclera, multiple fractures, Bilateral elbows and knee contractures and bilateral talipes, mild scoliosis, pectus carinatum.	19
			c.1559dupC, p.(Val523Cysfs*7)	41,424	14			
Egypt	1/1	Homozygous	c.1943G > C, p.(Arg648Pro)	3,769,394	18	Missense	White sclera, few fractures. Bilateral contractures at shoulders and right elbow and left knee. Anterior bowing (Saber) of tibia bilateral, Pectus excavatum visible prominent tailbone	19
Spain	2/1	Compound heterozygous	c.2122-2 A > G, p.?	2,576,839	19i	Splice site, Missense	Older sibling: White sclera, no joint contractures, mild bone fragility.	4
			c.1864G > T, p.(Gly622Cys)	1,479,469	18			
							Younger sibling: Blue-Grey sclera, bone fragility, recurrent fractures, cervical contracture (torticollis) during his first month of life	
Finnish, Italian, Eastern Europe	2/1	Compound heterozygous	c.1754 A > T, p.(Asp585Val)	3,363,157	17	Missense, Nonsense	Prenatal lethality, severe skeletal dysplasia, deformities, fractures	3
			c.497C > G, p.(Ser166*)	988,517	4			
European American	1/1	Compound heterozygous	c.797G > T, p.(Gly266Val)	2,076,023	8	Missense	Blue sclera, Wormian bones, recurrent fractures, cleft soft palate, scoliosis, bell-shaped thorax, pulmonary disease, congenital fusion of cervical vertebrae, dysmorphic features without joint contractures.	7
			c.1280 A > G, p.(Asn427Ser)	807,467	12	Missense		
Brazil	1/1	Homozygous	c.1682G > A, p.(Trp561*)	4,846,892	15	Nonsense	Respiratory difficulties, camptodactyly, mandibular hypoplasia; retrognathia; prominent eyes; broad nasal root; facial nevus flammeus; cleft palate; and skin dimples, fractures, deformities, ventricular septal defect. Died at 3m4d	3
Brazil	1/1	Compound heterozygous	c.1682G > A, p.(Trp561*)	4,846,892	16	Nonsense	Bruck syndrome (severe), NA	22
			c.1496_1500delAAATG, p.(Glu499Aspfs*29)	NA	13	Frameshift		
Canada	1/1	Compound heterozygous	c.1406G > A, p.(Gly469Glu)	1,685,013	13	Missense	Bruck syndrome, NA	21
			c.2154 T > A, p.(His718Gln)	NA	20	Missense		
United States	1/1	Compound heterozygous	c.2184_2187dup, p.(Met730Leufs*12)	NA	20	Frameshift	Bruck syndrome. Congenital elbow contracture, neonatal fractures	7
			c.1280 A > G, p.(Asn427Ser)	807,467	12	Missense		
United States	1/1	Homozygous	c.517G > C, p.(Ala173Pro)	1,392,586	5	Missense	Joint contractures, micrognathia, fractures, deformities, cubital tunnel syndrome	26
American (European)	2/1	Compound heterozygous	c.1958C > G, p.(Pro653Arg)	1,705,607	18	Missense, Nonsense	Older sibling: joint contractures, fractures, Wormian bones, clubfeet, plagiocephaly, hypoplasia.	2
			c.1318C > T, p.(Arg440*)	2,574,996	12			
							Younger sibling: fractures, no contractures, micrognathia, hypoplasia,	

NA – not available.

Genetically, BRKS is classified into two subtypes: Bruck syndrome type 1 (BRKS1, OMIM #259450) and Bruck syndrome type 2 (BRKS2, OMIM #609220). Although both types are clinically similar, BRKS1 is caused by pathogenic variants in the *FKBP10* gene (OMIM* 607063), while BRKS2 results from pathogenic variants in the *PLOD2* gene (OMIM* 601865).

The *PLOD2* gene encodes telopeptidyl lysyl hydroxylase 2 (LH2), an enzyme that catalyzes the hydroxylation of lysine residues to 5-hydroxylysine at the N- and C-terminal telopeptides of type I procollagen (Otaify et al., 2016; Brown and Zacharin, 2009). This post-translational modification is essential for the subsequent glycosylation of hydroxylysine and the formation of stable, irreversible intermolecular cross-links (Kaneto et al., 2016; Yamauchi and Sricholpech, 2012). LH2 exists in two isoforms: LH2a and LH2b. The LH2a isoform is the shorter transcript, whereas the LH2b isoform, encoded by transcript NM_182943.3 (the canonical MANE transcript) contains an additional 63-base pair exon (exon 13 A) that encodes 21 extra amino acids and is thought to be critical for its peptidyl hydroxylase activity (Zhang et al., 2021a). The hydroxylation process mediated by LH2 is vital for the mechanical strength and structural integrity of stiff connective tissues such as bone, ligaments, cartilage, and tendons (Terajima et al., 2022; Brown and Zacharin, 2009).

In this report, we present the first genetically confirmed cases of BRKS2 from Indonesia and Ukraine, thereby expanding the known geographic distribution of this ultra-rare congenital bone fragility disorder.

## 2 Case

### 2.1 Materials and methods

Clinical data were obtained through a review of medical records and patient-reported medical history. Physical examinations and phenotype documentation were carried out by OI-expert teams of the Universitas Diponegoro (Semarang, Indonesia) and the University of Tartu (Tartu, Estonia), part of the European Reference Network on Rare Bone Diseases (ERN BOND), in collaboration with the Institute of Spine and Joint Pathology (Kharkiv, Ukraine). This study was conducted in accordance with the principles of the Declaration of Helsinki, and all patients and/or families provided written informed consent prior to enrollment.

EDTA-blood was used to isolate the DNA of the Indonesian patient with the Genomic DNA Mini Kit (Geneaid, Cat. #GB100, Korea). DNA from the Ukrainian patient was isolated with the Gentra Puregene Blood Kit (Qiagen, Germany). Genetic analysis was performed at the Genome Diagnostics Laboratory of Amsterdam UMC, member of the European Reference Network on Rare Bone Diseases (ERN BOND). Targeted NGS analysis was performed using a custom-made kit enriched in OI related genes (KAPA HyperChoice (Roche) version CTDv3). Samples were sequenced with the Illumina MiSeq sequencing platform (Illumina, CA, USA) with coverage of at least 30×. NGS data were processed with an in-house bioinformatics analysis pipeline consisting of the Burrows-Wheeler Aligner for alignment of reads, the Genome Analysis Tool Kit Haplotype Caller for bam file processing and Alissa v.5 software (Agilent Technologies, CA, USA). NGS-based copy number variant (CNV) analysis was performed using read-depth based normalization of targeted regions within the in-house bioinformatics pipeline. The Alamut Visual v2.15 software (SOPHiA Genetics, Switzerland) was used to analyse the variant. Classification of variant pathogenicity was done in accordance with ACMG guidelines (Richards et al., 2015) with subsequent modification as described in The ClinGen Sequence Variant Interpretation Working Group (Group, T.C.S.V.I.W., 2019; Group, T.C.S.V.I.W., 2020; Pejaver et al., 2022; Tavtigian et al., 2020; Walker et al., 2023). The performed targeted NGS panel “Osteogenesis imperfecta and related disorders, version 3”, included 41 genes associated with OI and overlapping connective tissue disorders: NM_000478.6 (*ALPL),* NM_213599.3 (*ANO5),* NM_080605.4 (*B3GALT6),* NM_012200.4 (*B3GAT3),* NM_007255.3 (*B4GALT7),* NM_006129.5 (*BMP1*); NM_024821.5 (*CCDC134),* NM_004273.5 (*CHST3),* NM_000088.3 *(COL1A1),* NM_000089.4 (*COL1A2),* NM_052854.4 (*CREB3L1*); NM_006371.5 (*CRTAP*); NM_021939.4 (*FKBP10*); NM_152281.3 *(GORAB),* NM_001025295.3 (*IFITM5*); NM_006854.4 (*KDELR2),* NM_001127671.2 *(LIFR),* NM_002335.4 (*LRP5),* NM_015884.4 (*MBTPS2),* NM_015154.3 *(MESD),* NM_015909.4 *(NBAS)*, NM_022356.3 (*P3H1*); NM_000917.4 (*P4HA1),* NM_000918.4 (*P4HB*); NM_182943.3 (*PLOD2*); NM_001084.5 (*PLOD3),* NM_005032.7 *(PLS3*); NM_000942.5 (*PPIB*); NM_001318066.2 (*SEC24D*); NM_002615.7 (*SERPINF1*); NM_001235.5 (*SERPINH1*), NM_152621.6 (*SGMS2),* NM_001029998.6 (*SLC10A7),* NM_001173467.3 (*SP7*); NM_003118.4 (*SPARC*); NM_153365.5 (*TAPT1*); NM_017633.3 *(TENT5A),* NM_018112.3 (*TMEM38B*); NM_005430.4 (*WNT1*), NM_022166.4 (*XYLT1),* NM_022167.4 (*XYLT2*).

A comparative analysis of published cases in the literature and those presented in the current study was performed using descriptive statistical methods. The distribution of variants and associated phenotypic features was evaluated using percentage-based comparisons. Data were analysed using Microsoft Excel, and graphical representations were generated using the same software.

### 2.2 Case report 1

**Fig. 1:**
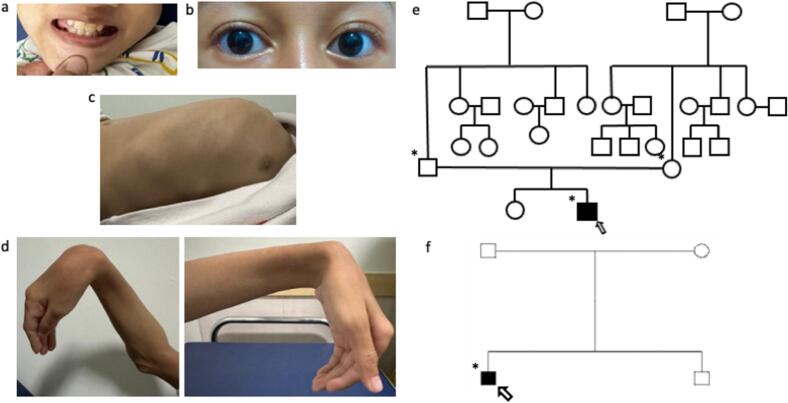
Clinical features and family pedigree tree of Case 1. (a) Dentinogenesis imperfecta. (b) Blue sclera. (c) Sternum and rib deformities, kyphosis. (d) Contractures of elbow and wrists. (e) Family pedigree of the patient from Case 1. (f) Family pedigree of the patient from Case 2. Arrow indicates proband, asterisk indicates genetic testing.

A 9-year-old Indonesian male presented to the Center for Biomedical Research, Faculty of Medicine, Universitas Diponegoro (Semarang, Indonesia) with a history of multiple fractures, skeletal deformities, and joint contractures. He was born to a 28-year-old mother (G2P1A0) following an uncomplicated and healthy pregnancy. No infections, complications, or use of unprescribed medications were reported during gestation. At birth, his weight was 2.8 kg and his length was 49 cm. The family is non-consanguineous, with no known history of genetic or hereditary disorders (Fig. 1).

The patient sustained his first fracture, a femoral break, at the age of one. Since then, he has experienced a total of eleven fractures, the most recent being an open humeral fracture at the age of eight. Clinical examination revealed blue sclera, DI, and bottle caries. There were no signs of facial dysmorphism, hearing loss, or visual impairment. Audiometric and tympanometry assessments confirmed normal hearing. Musculoskeletal evaluation showed elbow and wrist contractures, acetabular hypermobility with deformity, sternal and rib deformities, and kyphosis (Fig. 1). Additionally, the patient exhibited gross motor developmental delay, microcephaly (Z score − 2.81), severe underweight (Z score − 7.4), and short stature (Z score − 7.18), likely secondary to skeletal abnormalities and joint contractures.

The skeletal survey revealed generalized osteopenia, Wormian bones in the skull, spinal platyspondyly, acetabular protrusion, and multiple skeletal deformities. These included evidence of old fractures involving the right posterior 5th–9th ribs and the proximal to midshaft region of the left ulna.

**Fig. 2:**
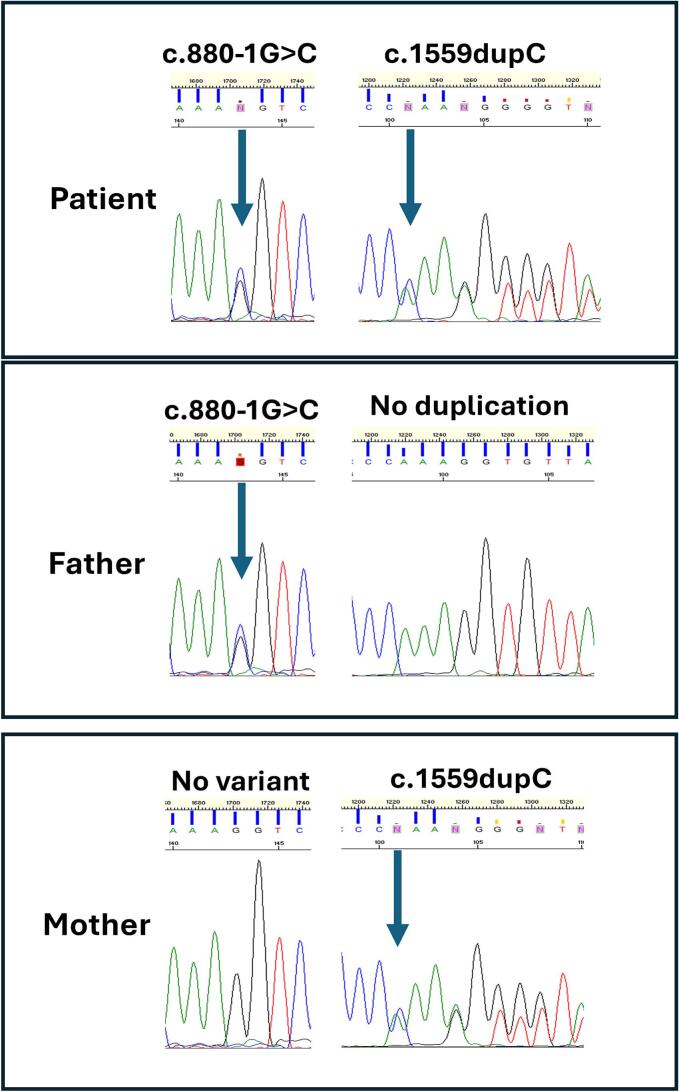
Sanger sequencing of *PLOD2* variants identified in the patient and parents of Case 1. Compound heterozygosity for these variants was confirmed by Sanger sequencing of the parents. The mother was a heterozygous carrier of the c.1559dupC variant, while the c.880-1G > C variant was identified in the father.

Genetic testing using the targeted next-generation sequencing (NGS) custom-made OI-related gene panel was performed. The analysis identified compound heterozygous NM_182943.3(PLOD2):c.880-1G > C, p.? and NM_182943.3(PLOD2):c.1559dupC, p.(Val523Cysfs*7) variants in the *PLOD2* gene (Fig. 2). Compound heterozygosity for these variants was confirmed by Sanger sequence analysis in the parents: the mother was a heterozygous carrier of the c.1559dupC variant, while the c.880-1G > C was identified in the father (Fig. 2).

The c.880-1G > C splice site variant affects the consensus sequence of intron 8 acceptor sites. As such, *in silico* this variant is predicted to abolish the acceptor site (Splice AI score for acceptor loss = 0.96, Splice-Altering / Strong). One potential effect of this variant is the skipping of the nearby located exon 9, leading to an in-frame deletion of 42 amino acids (p.Val294_Lys335del). This deletion concerns several amino acid residues predicted to undergo post-translational modifications, as reported in UniProt. The variant is absent in control population databases (https://gnomad.broadinstitute.org/v.4.1) and the OI Leiden Open Variation Database (LOVD) database, indicating it as a novel undescribed variant. This variant is classified as likely pathogenic according to American College of Medical Genetics and Genomics (ACMG) criteria (PVS1_Mod, PM2_Supp, PM3, PP4). Variant classification was performed using the MANE Select transcript NM_182943.3 (PLOD2), and ACMG criteria were applied according to standard guidelines, with PVS1 applied at a moderate level due to uncertainty in the predicted splicing effect.

The c.1559dupC is a frameshift variant in exon 14, introducing a premature stop codon. This variant has been previously reported in homozygous state in two index patients affected with BRKS2 (Puig-Hervas et al., 2012) and in compound heterozygosity with a missense variant in a patient with BRKS2 (Otaify et al., 2022). It is rare in population databases (Grpmax Filtering Allele Frequency (95% confidence) = 0.000018; gnomadAD Browser, v.4.1). It is classified as pathogenic based on ACMG guidelines (PVS1, PM2_Supp, PM3_Strong, PP4).

Based on clinical presentation and molecular findings, the patient was diagnosed with BRKS2. He has undergone multiple surgical corrections for fractures and began treatment with zoledronic acid at the age of four, alongside ascorbic acid, calcium lactate, and vitamin D₃ supplementation. At the age of eight, bone mineral density (BMD) scan showed a normal age-matched *Z*-score of 0.7 and total BMD of 0.689 g/cm^2^. Anthropometric measurements at the time included a weight of 13.4 kg, height of 95 cm, and BMI of 14.8 kg/m^2^.

### 2.3 Case report 2

The patient is a 4-year-old Ukrainian male, born into a non-consanguineous family with no known history of genetic disorders (Fig. 1). He was delivered after an uncomplicated pregnancy, with a birth weight of 3.17 kg and a length of 49 cm. An intrauterine fracture of the right hip was identified at birth, accompanied by congenital skeletal deformities. By the age of four, the patient had sustained more than 20 fractures.

Clinical evaluation revealed greyish sclerae and DI, but no hearing loss. Additional findings included a cleft palate, severe skeletal deformities of both upper and lower limbs, kyphoscoliosis, pectus excavatum, hypotrophy, and arthrogryposis. The patient had been previously diagnosed with Escobar syndrome based on clinical presentation.

Genetic testing with the targeted NGS custom-made OI-related gene panel identified a homozygous *PLOD2* variant, NM_182943.3(PLOD2): c.1958C > G, p.(Pro653Arg), located in exon 18. This missense variant results in the substitution of proline with arginine at position 653 within the prolyl 4-hydroxylase alpha subunit domain. The differences in chemical and physical properties between the small hydrophobic amino acid proline and the large and basic amino acid arginine are quite large (Grantham distance = 103). *In silico* prediction tools suggest a deleterious effect, with a REVEL score of 0.83. The affected proline residue is highly conserved across species (phyloP score: 9.87), suggesting functional importance.

This variant has been described in compound heterozygosity with a pathogenic stop codon variant in two siblings with BRKS2 (Gistelinck et al., 2021). Functional studies reported by Gistelinck et al. showed abnormal hydroxylation of type I collagen telopeptide lysine in the proband (Gistelinck et al., 2021). Also, this variant has been recorded in ClinVar (ID: 1705607) with conflicting interpretations of pathogenicity, and in at least one patient it has been associated with BRKS2 in homozygous state. It is also listed in dbSNP (rs967744523) but is absent from the OI LOVD database. This variant is very rare in population databases (Grpmax Filtering Allele Frequency (95% confidence) = 0.0000036; gnomadAD Browser, v.4.1). According to ACMG guidelines, the variant is classified as likely pathogenic based on criteria PM2_Supp, PP3_Mod, PM3_Strong, PP4.

Based on clinical features and genetic findings, a definitive diagnosis of BRKS2 was established. The patient was treated with pamidronic acid, calcium, and vitamin D₃.

## 3 Discussion and literature review

We present two cases of BRKS2-associated skeletal dysplasia characterized by recurrent bone fractures, severe skeletal deformities, and upper limb contractures (Fig. 1). The overall clinical presentation in both cases was consistent with previously reported BRKS2 cases (Caparros-Martin et al., 2017; Lv et al., 2018), showing hallmark features such as frequent fractures, skeletal deformities comparable to moderate to severe OI, and joint contractures.

Previous literature described 44 patients from 35 unrelated families with biallelic *PLOD2* variants (Table 1, Table 2, Supplementary Fig. S1) (Gistelinck et al., 2021; Leal et al., 2018; Puig-Hervas et al., 2012; Caparros-Martin et al., 2017; Lv et al., 2018; Mumm et al., 2020; Zhang et al., 2021a; Otaify et al., 2022; Arora et al., 2018; Bardai et al., 2016; Fernandes et al., 2020; Ha-Vinh et al., 2004; Saini et al., 2022; Sandy et al., 2023; Santana et al., 2019; van der Slot et al., 2003; Zhang et al., 2021b). Homozygous cases represented 57% (20/35), and the remaining cases were compound heterozygous. Most variants were missense (41/70, 58.6%) from all alleles, followed by nonsense (10/70, 14.3%) and frameshift (10/70, 14.3%) variants, splice-site variants (7/70, 10%), and one large homozygous deletion (2/70, 2.9%). Variants were non-uniformly distributed across the 20 coding exons of *PLOD2* and showed pronounced clustering within the C-terminal Fe^2+^/2-oxoglutarate-dependent dioxygenase domain (encoded by exons 17–20) that carries the lysyl-hydroxylase 2 catalytic activity (Guo and Kurie, 2018; Wang et al., 2022). Exon 18 alone harbored 28.6% (20/70) alleles and was dominated by missense substitutions, representing a previously suggested mutational hotspot. Recurrent mutations included p.(Arg619His), p.(Pro653Arg), p.(Val523Cysfs*7) (founder allele), and p.(Asn427Ser) (Gistelinck et al., 2021; Puig-Hervas et al., 2012; Mumm et al., 2020; Walker et al., 2023; Otaify et al., 2022). Null-allele variants were scattered across the N-terminal and central parts of the protein (exons 2, 4, 6, 10, 15, 16, 19, 20), consistent with a null-allele effect.

Variant type uncovered possible trends in correlation with clinical severity. Individuals with null-allele variants exhibited the most severe phenotypes, with 50% (5/10 families) lethal outcomes (Table 1, Table 2, Supplementary Fig. S1). Biallelic missense variants (16 families) were generally compatible with survival. Only one family (Australian, homozygous p.(Thr629Ile)) reported pregnancy or early-infant loss, whereas the remaining patients presented classical, compatible-with-life BRKS2 with graded severity (van der Slot et al., 2003). Missense and null-allele compound heterozygotes (9 families) showed intermediate severity. Within the biallelic missense group, the position of the substitution appeared to associate with some phenotype patterns. Missense variants falling inside the catalytic dioxygenase domain (exons 17–20) were associated with the full BRKS2 phenotype: congenital joint contractures, recurrent fractures, progressive kyphoscoliosis and Wormian bones (Table 1). In contrast, missense variants outside or at the margins of the catalytic core tended to produce milder, sometimes contracture-free presentations. Intrafamilial variability was nonetheless notable, indicating that the genotype alone is insufficient to predict the severity of individual symptoms. Across 32 well-characterized families, recurrent fractures (97%), skeletal deformities (88%), and joint contractures (81%) were most common, followed by kyphoscoliosis, abnormal sclera color, and Wormian bones. Less frequent features included clubfoot, camptodactyly, cleft palate, and early lethality.

Taken together, comparison of all variants reported in the literature alongside those presented in our study supports that the nature of the variant type may affect overall severity and lethality risk, and that the protein-domain context of missense variants modulates the presence and severity of contractures and skeletal deformities.

**Table 2:** Summary of genetic and clinical landscape of patients with Bruck Syndrome type 2 (BRKS2) and (likely) pathogenic variants in the *PLOD2* gene (NM_182943.3) reported in the literature.

Size of the cohort	35 families
	44 patients
Variant zygosity n, (%)	Homozygous - 20/35 (57%)
	Compound heterozygous - 15/35 (43%)
Variant types n, (%) (allele-level, *n* = 70)	Missense - 41 (58.6%)
	Nonsense - 10 (14.3%)
	Frameshift - 10 (14.3%)
	Splice site - 7 (10.0%)
	Large Deletions - 2 (2.9%)
Exon hotspots n, (%) (allele-level, n = 70)	Exon 18–20 alleles (28.6%)
	Exon 12–8 alleles (11.4%)
	Exon 14–6 alleles (8.6%)
	Exon 17–5 alleles (7.1%)
	Exon 19–5 alleles (7.1%)
Recurrent variants	c.1856G > A, p.(Arg619His) or c.1856G > T, p.(Arg619His): 4 families (1 Turkish, 3 Egyptian)
	c.1559dupC, p.(Val523Cysfs*7): 4 families (Egyptian)
	c.1280 A > G, p.(Asn427Ser): 3 families (transcontinental)
	p.(Pro653Arg), p.(Gly266Val), p.(Arg680*), p.(Trp561*): 2 families each
Trends in genotype severity	*Variant type based*
	Biallelic loss-of-function - 50% lethal (5/10)
	Biallelic missense - 6% lethal (1/16; homozygous p.(Thr629Ile))
	Missense and null-allele - 11% lethal (1/9), most severe-to-moderate
	*Variant position based*
	Catalytic-domain missense (exons 17–20) - classical severe BRKS2
	Non-catalytic alleles - milder, often contracture-free
Phenotypic trends n, (%) (32 characterized families)	Recurrent fractures 31/32 (97%)
	Skeletal deformities 28/32 (88%)
	Congenital contractures 26/32 (81%)
	Kyphoscoliosis 15/32 (47%)
	Blue/grey sclerae 13/32 (41%)
	White sclerae 11/32 (34%)
	Wormian bones 10/32 (31%)
	Clubfoot/talipes 7/32 (22%)
	Prenatal/early lethal 6/32 (19%)
	Camptodactyly 5/32 (16%)
	Cleft palate 3/32 (9%)

There was variability in the phenotypic severity between the two patients. The Ukrainian patient exhibited a more severe presentation, including intrauterine fractures, while the Indonesian patient experienced the first fracture only at one year of age. Similar variability has been reported previously, not only between unrelated individuals with different pathogenic variants, but also among siblings within the same family. Some BRKS2 patients have also been reported to lack one of the key features, joint contractures, highlighting the broad clinical spectrum of the disorder (Gistelinck et al., 2021; Leal et al., 2018; Puig-Hervas et al., 2012). However, a limitation of this study is the lack of available radiographic images, which precluded detailed assessment of skeletal deformities, vertebral abnormalities, fracture healing patterns, and long bone morphology.

Differences in the degree of bone fragility and deformity have also been noted across patients (Table 1). Although BRKS2 patients are typically described as lacking blue sclerae, hearing loss, and DI, both of our patients exhibited signs of DI. Orodental anomalies have been previously reported, including enamel defects, microdontia, and a high-arched palate, as observed in the cohort studied by Otaify et al (Otaify et al., 2022). The Ukrainian patient had greyish sclerae, while the Indonesian patient had blue sclerae. Variations in scleral hue, including greyish or bluish tones, have been reported, although these do not appear to correlate with genotype, as differences can occur even within the same family. Wormian bones were observed in the Indonesian BRKS2 patient in our report and have also been documented in American, Egyptian, and Spanish patients previously (Gistelinck et al., 2021; Puig-Hervas et al., 2012; Caparros-Martin et al., 2017; Mumm et al., 2020).

Additionally, the Ukrainian patient in our study presented with cleft palate, a feature previously reported in a Brazilian patient with a nonsense *PLOD2* variant. Arched palates have also been noted by Otaify et al (Otaify et al., 2016). As more cases of this ultra-rare disorder are documented, it may become possible to establish clearer genotype–phenotype correlations, similar to those in OI, which could advance our understanding of the underlying pathophysiology and variability of the disorder.

Both patients received bisphosphonate therapy in combination with corrective orthopaedic surgeries. Bisphosphonates are widely used to manage bone fragility in moderate to severe OI, and recent studies suggest they offer similar benefits in BRKS, including reduced fracture rates, decreased pain levels (Otaify et al., 2016), and improved BMD (Brown and Zacharin, 2009). In Case 1, initial radiographs revealed a porotic bone structure, which normalized following four years of treatment with zoledronic acid, as confirmed by BMD scanning.

In Case 1, genetic analysis revealed compound heterozygous variants in the *PLOD2* gene. The first is a novel splice-site mutation (c.880-1G > C), which has not been previously reported in public variant databases. The identified variant is consistent with aberrant splicing as a potential pathogenic mechanism in *PLOD2*-related cases and suggests the possibility that partial loss-of-function effects may contribute to the BRKS2 phenotype. *In silico* analyses predict that the variant disrupts the canonical acceptor site, potentially resulting in abnormal splicing. One possible consequence is exon skipping, leading to an in-frame deletion of 42 amino acids. *In silico* analyses also predict activation of two potential cryptic acceptor splice sites, which could result in an out-of-frame deletion and generation of a null-allele. However, this latter scenario may be less likely, as complete loss-of-function variants in *PLOD2* have been proposed to be incompatible with life (Leal et al., 2018; Hyry et al., 2009).

Importantly, these predicted molecular consequences have not been experimentally validated. As the pathogenicity of the novel splice-site variant is currently based on *in silico* predictions, its precise effect on splicing remains uncertain and represents a limitation of this study, requiring further RNA-based functional validation. Identification of a novel pathogenic splice site variant with predicted pathogenic effects expands the mutational spectrum of *PLOD2* and highlights the importance of comprehensive genetic analysis in patients with unexplained bone fragility.

The second variant, a known pathogenic frameshift mutation (c.1559dupC), is predicted to introduce a premature termination codon, which is expected to cause a null-allele due to nonsense-mediated mRNA decay (NMD), or lead to the production of truncated LH2b protein fragments. This frameshift mutation is located in the alternative exon (13a), which is transcribed and translated only in the LH2b isoform. Therefore, this variant will specifically inactivate the LH2b isoform, which is essential for the hydroxylation of peptidyl lysines in collagen telopeptides, while it will have no effect on the LH2a isoform (Puig-Hervas et al., 2012; Zhang et al., 2021a; Hyry et al., 2009).

We previously reported a case of an Indonesian BRKS1 patient with variants in the *FKBP10* gene. The patient's phenotype was similar to that of an Indonesian BRKS2 patient presented in this study, with features such as joint contractures, fractures, and blue sclera. However, no DI was observed. BRKS1 and BRKS2 patients share overlapping phenotypes, as variants in both genes have a comparable impact on hydroxylation of pro-collagen type I telopeptides (Setijowati et al., 2012). Although BRKS1/2 are genetically distinct from OI, both disorders can present overlapping features, including bone fragility, blue sclera, and DI. The occurrence of blue sclera and DI in our Indonesian patient underscores this phenotypic overlap and emphasizes the importance of molecular testing for an accurate diagnosis.

Both types of BRKS are characterized by a decrease in the ratio of urinary collagen degradation products, specifically hydroxylysylpyridinolines (HP) and lysylpyridinolines (LP), to hydroxyproline (Hyp). The pyridinoline cross-links HP and LP are exclusively derived through the hydroxyallysine pathway and serve as indicators of telopeptide lysyl hydroxylation. This (HP + LP) to Hyp urine ratio has proven to be a valuable diagnostic marker for BRKS, providing an efficient and reliable method for screening (Ha-Vinh et al., 2004; Setijowati et al., 2012). This tool might be useful in the cohort of OI with contractures, facilitating early diagnosis and intervention for affected individuals.

**Fig. 3:**
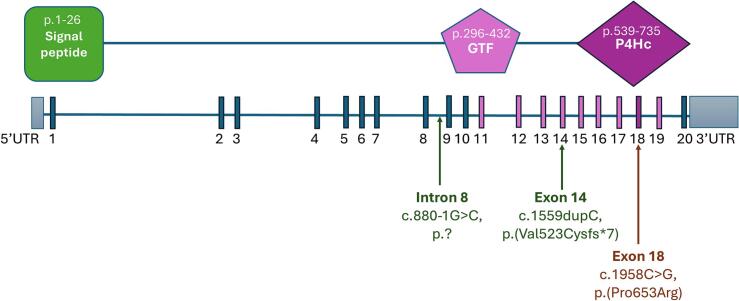
Structure of the *PLOD2* gene and protein product highlighting mutation hotspots (exons 11–19, and particularly exon 18), which overlap with the glucosyltransferase (GTF) and Prolyl 4-hydroxylase alpha subunit homolog (P4Hc) domains. The compound heterozygous pathogenic variants in the Indonesian BRKS2 patient (Case 1) are shown in green, while the homozygous pathogenic variant in the Ukrainian BRKS2 patient (Case 2) is depicted in orange.

Case 2 harbors a previously reported missense variant (c.1958C>G p.(Pro653Arg)), which has been described in compound heterozygosity with a nonsense variant known to trigger NMD (Gistelinck et al., 2021). Also, homozygosity for this missense variant has been reported in ClinVar. The patient in ClinVar has been described as having multiple prenatal fractures, clubfoot, knee flexion contracture, increased susceptibility to fractures, mild global developmental delay, abnormal facial shape (submitter: 3billion. ClinVar ID: 1705607). The variant is located in exon 18, previously suggested as a mutational hotspot (former exon 17, transcript NM_000935.3) (Ha-Vinh et al., 2004), and overlapping with a glucosyltransferase (GTF) and Prolyl 4-hydroxylase alpha subunit homolog domain (P4Hc) of the protein (Fig. 3) (Qi and Xu, 2018).

Even though no functional studies were performed in this study to evaluate the molecular effects of the identified variants, previous functional studies in patients with compound heterozygous *PLOD2* mutations revealed that type I collagen showed markedly reduced hydroxylation of telopeptide lysines, while triple-helical lysine hydroxylation remained normal. This defect led to a near-complete loss of stable trivalent cross-links and the predominance of allysine-derived aldol dimeric cross-links, a pattern typically seen in skin collagen. Type II collagen peptides from urine displayed normal telopeptide lysine hydroxylation. This contrast highlights the tissue-specific nature of lysyl hydroxylation and cross-linking, emphasizing the critical role of LH2b in bone collagen maturation. The selective disruption of bone collagen cross-linking likely underlies the severe skeletal phenotype observed in these patients, explaining the compromised mechanical integrity of the extracellular matrix, which is consistent with the OI pathophysiology (Gistelinck et al., 2021). Further understanding of the LH2b and LH2a isoform-specific disruption may inspire future therapeutic strategies, including isoform-targeted gene therapy and small molecules that enhance residual LH2b function.

Impaired collagen cross-linking due to defective LH2 activity remains central to the pathophysiology of BRKS. Pathogenic variants in the *PLOD2* gene, which encodes LH2, disrupt the hydroxylysine component of collagen cross-linking, leading to compromised connective tissue stability (Gistelinck et al., 2021; Hyry et al., 2009; Gistelinck et al., 2016).

We report the first documented cases of BRKS2 in Indonesia and Ukraine, involving homozygous and compound heterozygous *PLOD2* pathogenic variants. These findings expand the phenotypic and geographic spectrum of BRKS2 and underscore the importance of molecular diagnostics in establishing a definitive diagnosis, informing management strategies, and enabling genetic counseling.

Further research is needed to deepen our understanding of *PLOD2*-related disease mechanisms and to explore potential therapeutic targets. Longitudinal studies and the development of patient registries will be critical for characterizing the natural history of BRKS2 and identifying modifiers of disease severity and treatment response.

## CRediT authorship contribution statement

**Devina Afraditya Paveta:** Writing – original draft, Visualization, Investigation, Formal analysis. **Agustini Utari:** Writing – review & editing, Supervision, Project administration, Methodology, Investigation, Funding acquisition, Conceptualization. **Ferdy Kurniawan Cayami:** Methodology, Investigation. **Alessandra Maugeri:** Validation, Methodology, Formal analysis. **Aare Märtson:** Resources, Methodology, Investigation. **Sulev Kõks:** Resources, Methodology, Investigation. **Andrii Pashenko:** Resources, Methodology, Investigation. **Sergii Khmyzov:** Resources, Methodology, Investigation. **Elisabeth Marelise W. Eekhoff:** Resources, Methodology, Investigation. **Katre Maasalu:** Project administration, Methodology, Investigation, Funding acquisition, Formal analysis, Conceptualization. **Dimitra Micha:** Writing – review & editing, Project administration, Methodology, Investigation, Formal analysis, Conceptualization. **Lidiia Zhytnik:** Writing – review & editing, Writing – original draft, Visualization, Project administration, Methodology, Investigation, Formal analysis, Conceptualization.

## Ethical form

Written informed consent was obtained from all patients and/or their legal guardians. The study was conducted in accordance with the principles of the Declaration of Helsinki and received ethical approval from the Health Research Ethics Committee, Faculty of Medicine, Universitas Diponegoro (No.389/EC/KEPK/FK UNDIP/VIII/2019) and the Ethical Review Committee on Human Research at the University of Tartu (No. 221/M-34).

## Declaration of competing interest

The authors declare that they have no known competing financial interests or personal relationships that could have appeared to influence the work reported in this paper.

## Data Availability

Data will be made available on request.

## Appendix A Supplementary data

Supplementary Fig. S1 Genotype-phenotype landscape of *PLOD2* in Bruck Syndrome type 2 (BRKS2).
