# Supplementary material for: Extending the global landscape of Bruck syndrome: Case series of Indonesian and Ukrainian patients with PLOD2 pathogenic variants and literature review
Source: Bone Rep. 2026 Jul 7;30:101938. doi: 10.1016/j.bonr.2026.101938 (PMC13380098; doi:10.1016/j.bonr.2026.101938)
Supplement: Supplementary Fig. S1 — Genotype-phenotype landscape of PLOD2 in Bruck Syndrome type 2 (BRKS2). [file mmc1.docx]

**Supplementary Figure S1.** Genotype-phenotype landscape of *PLOD2* in Bruck Syndrome type 2 (BRKS2).


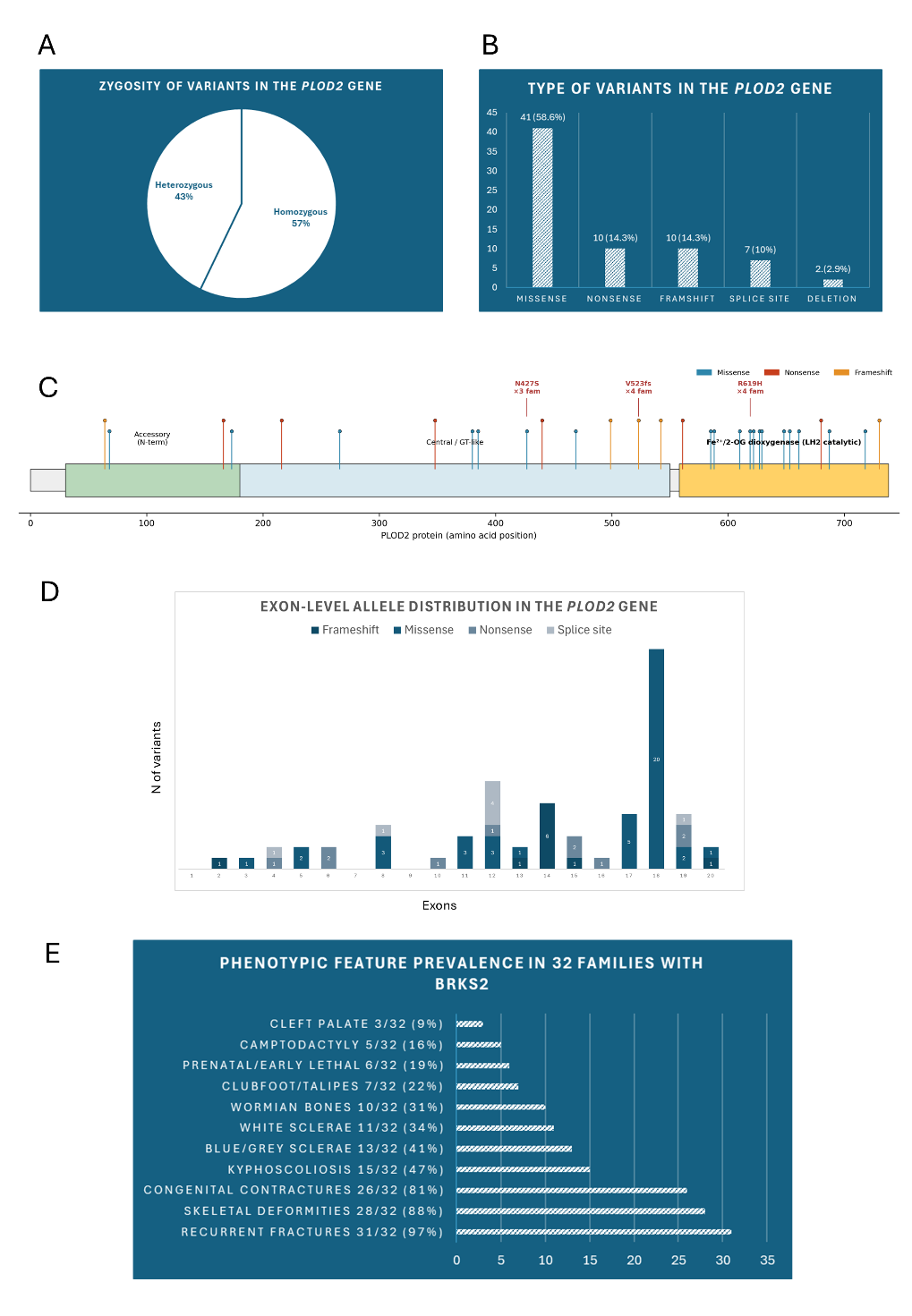


(A) Zygosity distribution at the family level. (B) Allele-level distribution of the 70 reported variants by variant type. (C) Mapping of all reported protein-level variants to the PLOD2 protein, showing clustering of missense substitutions within the C-terminal Fe²⁺/2-oxoglutarate-dependent dioxygenase (LH2 catalytic) domain. (D) Exon-level allele count (intronic canonical splice sites pooled with their flanking exon). Exon-18 is the major hotspot. Exons 12 and 14 carry recurrent variants. (D) Frequency of major clinical features across the 32 characterized families with detailed phenotype data.
